# Copper-enriched automotive brake wear particles perturb human alveolar cellular homeostasis

**DOI:** 10.1186/s12989-024-00617-2

**Published:** 2025-02-13

**Authors:** James G. H. Parkin, Lareb S. N. Dean, Joseph A. Bell, Natasha H. C. Easton, Liam J. Edgeway, Matthew J. Cooper, Robert Ridley, Franco Conforti, Siyuan Wang, Liudi Yao, Juanjuan Li, Helen Vethakan Raj, Julian Downward, Miriam Gerlofs-Nijland, Flemming R. Cassee, Yihua Wang, Richard B. Cook, Mark G. Jones, Donna E. Davies, Matthew Loxham

**Affiliations:** 1 School of Clinical and Experimental Sciences, University of Southampton, Southampton, UK; 2 NIHR Southampton Biomedical Research Centre, University Hospital Southampton, Southampton, UK; 3 Southampton Marine and Maritime Institute, University of Southampton, Boldrewood Innovation Campus, Southampton, UK; 4 School of Ocean and Earth Sciences, University of Southampton, Southampton, UK; 5 Institute for Life Sciences, University of Southampton, Highfield Campus, Southampton, UK; 6 Biological Sciences, Faculty of Environmental and Life Sciences, University of Southampton, Southampton, UK; 7 Oncogene Biology Laboratory, The Francis Crick Institute, London, UK; 8 National Institute for Public Health and the Environment (RIVM), Bilthoven, Netherlands; 9 Institute for Risk Assessment Sciences (IRAS), Utrecht University, Utrecht, The Netherlands; 10 National Centre for Advanced Tribology (nCATS), Mechanical Engineering, Faculty of Engineering and Physical Sciences, University of Southampton, Southampton, UK

**Keywords:** Brake-wear PM, Non-exhaust emissions, Non-tailpipe emissions, Alveolar, Epithelium, Pseudohypoxic signalling, Hypoxia-inducible factor (HIF), Metallothionein, Diesel PM, Copper

## Abstract

**Background:**

Airborne fine particulate matter with diameter < 2.5 μm (PM2.5), can reach the alveolar regions of the lungs, and is associated with over 4 million premature deaths per year worldwide. However, the source-specific consequences of PM2.5 exposure remain poorly understood. A major, but unregulated source is car brake wear, which exhaust emission reduction measures have not diminished.

**Methods:**

We used an interdisciplinary approach to investigate the consequences of brake-wear PM2.5 exposure upon lung alveolar cellular homeostasis using diesel exhaust PM as a comparator. This involved RNA-Seq to analyse global transcriptomic changes, metabolic analyses to investigate glycolytic reprogramming, mass spectrometry to determine PM composition, and reporter assays to provide mechanistic insight into differential effects.

**Results:**

We identified brake-wear PM from copper-enriched non-asbestos organic, and ceramic brake pads as inducing the greatest oxidative stress, inflammation, and pseudohypoxic HIF activation (a pathway implicated in diseases associated with air pollution exposure, including cancer, and pulmonary fibrosis), as well as perturbation of metabolism, and metal homeostasis compared with brake wear PM from low- or semi-metallic pads, and also, importantly, diesel exhaust PM. Compositional and metal chelator analyses identified that differential effects were driven by copper.

**Conclusions:**

We demonstrate here that brake-wear PM may perturb cellular homeostasis more than diesel exhaust PM. Our findings demonstrate the potential differences in effects, not only for non-exhaust *vs* exhaust PM, but also amongst different sources of non-exhaust PM. This has implications for our understanding of the potential health effects of road vehicle-associated PM. More broadly, our findings illustrate the importance of PM composition on potential health effects, highlighting the need for targeted legislation to protect public health.

**Supplementary Information:**

The online version contains supplementary material available at 10.1186/s12989-024-00617-2.

## Background

Air pollution is the leading environmental risk factor for morbidity and mortality globally, with the World Health Organisation estimating that exposure to outdoor and indoor air pollution is associated with around 7 million premature deaths per year [1, 2]. A key air pollutant is particulate matter (PM)—a complex mixture of particles and liquid droplets suspended in the air, which has diverse physical and chemical properties [1]. The lungs are the primary site of PM deposition; fine PM (aerodynamic diameter < 2.5 µm; PM2.5) can deposit in the bronchioles and the alveoli, with some particles small enough to translocate into the bloodstream and elicit systemic effects [3]. Out of all air pollutants, exposure to fine PM has been associated with the largest impact on human life expectancy as well as all-cause and cause-specific mortality [4]. Currently, PM emissions are primarily regulated by mass concentration limits for a given size fraction (e.g. PM2.5), which assumes that all sources of these particulates exert the same biological effects.

A major source of PM2.5 in urban areas is road vehicles. Exhaust emissions (particularly diesel exhaust PM) have been the focus of extensive research [5–7]. As a consequence, exhaust emissions have decreased over time due to advances in emissions reduction technologies such as efficient diesel particulate filters [8, 9], with many governments implementing legislation changes targeting the reduction of exhaust emissions [9]. However, vehicles also generate PM from the wear of the road, tyres, and brakes, collectively termed ‘non-exhaust emissions’, with brake wear contributing up to 55% by mass [10, 11]. Non-exhaust emissions have increased commensurately with traffic, and now contribute more by mass than exhaust emissions to total vehicular PM2.5 in many European countries [9]. This trend is predicted to increase over time as there is a shift towards heavier battery electric vehicles, which generate more of these friction-derived, non-exhaust emissions [9, 12]. Currently, non-exhaust emissions are largely unregulated by legislation, meaning that there is a lack of established technologies to mitigate their release. They also tend to be more chemically heterogeneous than exhaust emissions, meaning that they may have the potential to elicit different biological effects depending on the source material [13, 14]. Together this points to an urgent and unmet need to better understand the source-specific biological effects of non-exhaust emissions, and more specifically – brake wear.

Brake-wear PM is generated via friction between the brake pad and disc, with its physicochemical properties being dependent upon the brake pad material, and is typically characterised by the presence of d-block/transition metals (including iron, copper, and zinc) [15]. Brake pads are formed from a combination of fillers, binders, abrasives, lubricants, and reinforcing fibres, which impart various speed-reduction, anti-fade, and noise-reduction properties. However, the exact combination of constituent components used in a brake pad formulation is not subject to regulation, with brake pads having distinct compositions within four classes: low-metallic (LowM), semi-metallic (SemiM), non-asbestos organic (NAO), or ceramic [16]. NAO brake pads are the most commonly used pad type in the US market due to their low cost, low noise, and relatively low wear rate [17, 18]. These pads were developed as a replacement for asbestos-containing brake pads, and have copper fibres added to the formulation to increase thermal conductivity, a property that would have been provided by the asbestos [17].

Here, we characterised the consequences of automotive brake-wear PM2.5 exposure upon lung alveolar homeostasis compared to diesel exhaust PM. We focused on alveolar type-II epithelial (ATII) cells, which are a important site of PM2.5 deposition and effect. These cells play a vital role in the response to alveolar injury, in particular, acting as a progenitor pool, able to differentiate to replace damaged ATI cells [19]. Repeated damage to ATII cells by PM2.5 has been linked to the initiation and progression of numerous diseases, including pulmonary fibrosis and pulmonary adenocarcinoma [20–24]. Therefore, through an inter-disciplinary approach combining chemical characterisation, transcriptomics, and toxicity studies we identified brake pad type-specific effects upon ATII cells, with the greatest effects defined by PM from pads with copper enrichment. This study highlights the potential for adverse respiratory and systemic effects of specific vehicle-derived PM types. This has implications for understanding the health impacts of road vehicle emissions, even after the upcoming decarbonisation of the road vehicle fleet.

## Results

### Characterisation of the alveolar responses to vehicle-derived PM

**Fig. 1:**
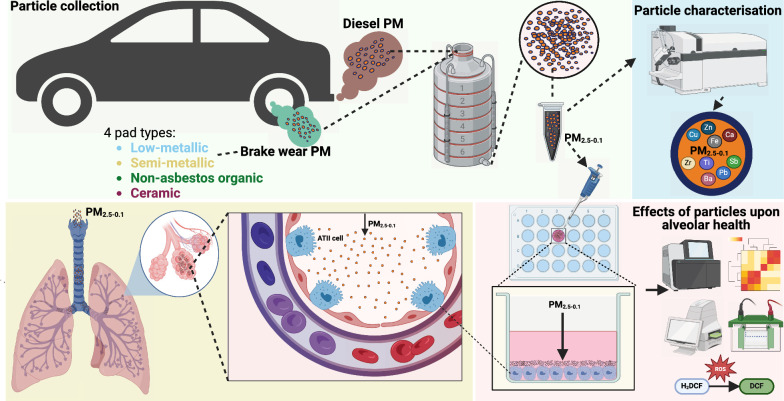
Overview of the study. Brake-wear PM from four different brake pad types, as well as diesel exhaust PM were collected in a laboratory setting using a high-volume cascade impactor onto foam filters. Foam filters were dried under nitrogen gas and PM was extracted using methanol. The composition of these PM were assessed using ICP-MS. Given that PM2.5–0.1 can reach the alveoli, we investigated the differential effects of the vehicle-derived PM types in a submerged culture of alveolar type-II epithelial cells. Designed using BioRender.com

**Fig. 2:**
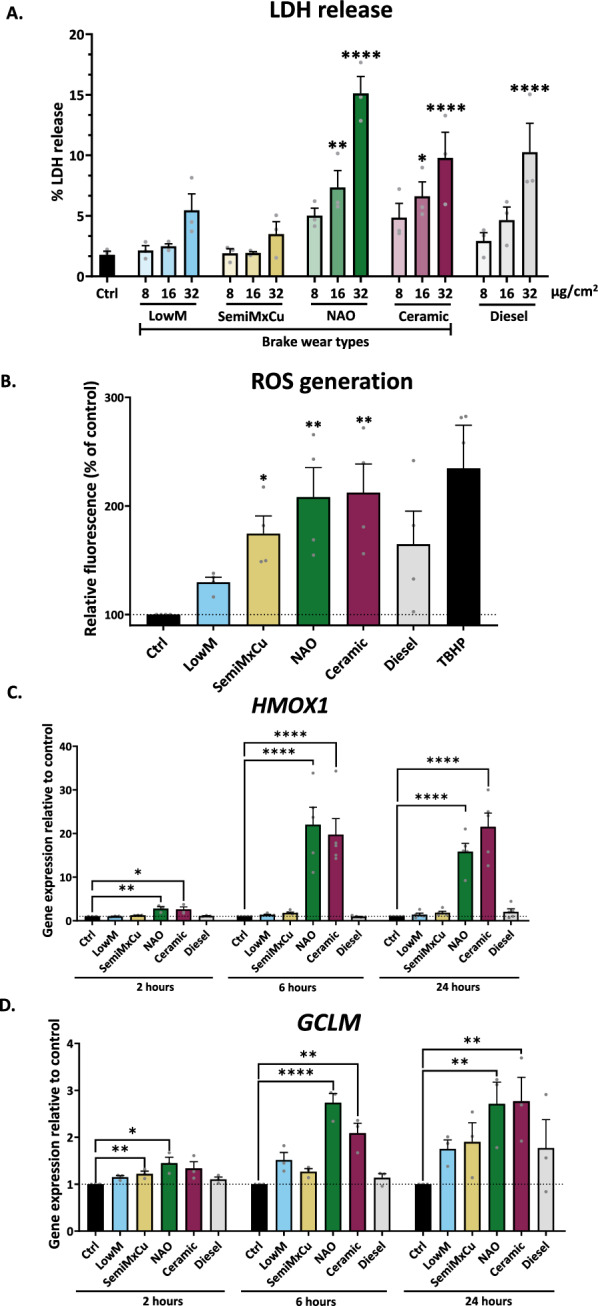
NAO and Ceramic brake-wear PM induce the greatest oxidative stress. ATII cells were exposed to 8, 16, and 32 µg/cm^2^ of the 5 different vehicle-derived PM types for 24 h. **A** LDH release was measured, with higher LDH release indicating higher cytotoxicity; LDH release was determined using a CytoTox 96^®^ Non-Radioactive Cytotoxicity Assay kit, n = 3. **B** ROS generation was assessed in ATII cells via oxidation of H_2_-DCF into the fluorescent DCF after 24 h of exposure to PM; tert-Butyl hydroperoxide (TBHP) was used as a positive control for ROS induction, n = 5. **C** ATII Haem Oxygenase-1 (*HMOX1*) expression was determined using RT-qPCR after exposure to 8 µg/cm^2^ of the 5 different PM types, for 2, 6 and 24 h, n = 3 for 2 h exposure. n = 5 for 6 and 24 h exposure. **D** ATII cell Glutamate-Cysteine Ligase Modifier Subunit (*GCLM*) expression was determined using RT-qPCR after exposure to 8 µg/cm^2^ of the 5 different PM types, for 2, 6 and 24 h, n = 3. Data was represented as mean + SEM, and a RM one-way ANOVA test was used with a Dunnett’s post-hoc test to determine significance compared to the control. * = *p* ≤ 0.05, ** = *p* ≤ 0.01, **** = *p* ≤ 0.0001

An overview of the study design is shown in Fig. 1. We studied PM2.5–0.1 (aerodynamic diameter 2.5–0.1 μm) from automotive brake-wear PM generated from four pad types (low-metallic, LowM; semi-metallic with limited copper, SemiMxCu; non-asbestos organic, NAO; and ceramic), using diesel exhaust PM2.5–0.1 as a comparator. To investigate the biological responses to each PM type, we first exposed an alveolar type-II epithelial cell line (ATII) to PM at concentrations up to 32 µg/cm^2^ for 24 h and assessed for cytotoxicity measured by lactate dehydrogenase (LDH) release. A significant concentration-dependent increase in cytotoxicity was observed following exposure to PM2.5–0.1 from NAO and ceramic brake-wear PM, and with diesel PM at 32 µg/cm^2^ (Fig. 2A). As no significant increase in cytotoxicity was observed in response to any of the PM types at 8 µg/cm^2^, this concentration was used for subsequent experiments.

We next looked for evidence of oxidative stress following exposure to PM for 24 h, using the reactive oxygen species (ROS)-sensitive dye dichlorofluorescein (DCF). NAO and ceramic brake-wear PM induced the greatest increase in ROS generation, while no significant effect was observed with an equivalent concentration of LowM or diesel PM (Fig. 2B). Consistent with this, analysis of antioxidant gene expression identified that *haem-oxygenase-1* (*HMOX1*) and *glutamate-cysteine ligase modifier subunit* (*GCLM*) were significantly upregulated in response to NAO and ceramic brake-wear PM, whereas no significant change in expression was seen in response to any of the other PM types (Fig. 2C, D). We confirmed similar results using primary ATII cells, where NAO and ceramic brake-wear PM led to the greatest increase in both *HMOX1* and *GCLM* expression (Fig. S1A, B). Together these results indicate differential cell responses to specific brake-wear PM types, with NAO and ceramic brake-wear PM inducing oxidative stress to the greatest extent.

**Fig. 3:**
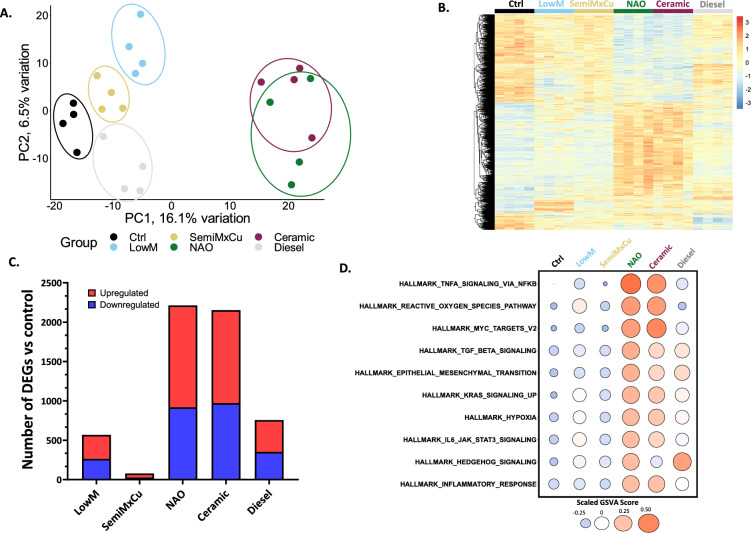
Transcriptomic responses to PM are source-dependent, with NAO and Ceramic inducing the greatest number and magnitude of changes. ATII cells were exposed to 8 µg/cm^2^ of the 5 different vehicle-derived PM types for 6 h, after which bulk RNA-Seq was conducted. The differential gene expression package edgeR was used to make pairwise comparisons between each of the five PM types and the control (e.g. LowM *vs* Ctrl, SemiMxCu *vs* Ctrl etc.…) to identify genes that were up or downregulated compared to the medium-only control. Genes with a false-discovery rate (FDR) *p*-value of ≤ 0.05 were considered differentially expressed. **A** Biplot showing the first two principal components, demonstrating that NAO and ceramic brake-wear PM are separated on PC1. **B** Heatmap of all genes that were differentially expressed in at least one of the 5 different pairwise comparisons, with hierarchical clustering of genes, generated using the ‘pheatmap’ package. **C** Number of DEGs for each PM type compared to the medium-only control. **D** Bubble plot showing Gene Set Variation Analysis (GSVA) scores for the top 10 most upregulated hallmark pathways by NAO brake-wear PM

Given our identification of type-specific brake-wear PM responses, we further investigated the impact of exposure of ATII cells to each PM type, using RNA-Seq as an untargeted approach to measure gene expression. Principal component analysis (PCA) identified overlap between the NAO and ceramic brake-wear PM-modulated gene expression profiles, whilst all other PM types were distinct (Fig. 3A). Differential gene expression analysis (Fig. 3B) identified that NAO and ceramic brake-wear PM induced the greatest number of differentially expressed genes (DEGs; defined by a false discovery rate-adjusted (FDR) p-value ≤ 0.05) (2212 and 2153 DEGs respectively), when compared to LowM (569 DEGs), SemiMxCu (78 DEGs) or diesel exhaust PM (767 DEGs) (Fig. 3C).

**Fig. 4:**
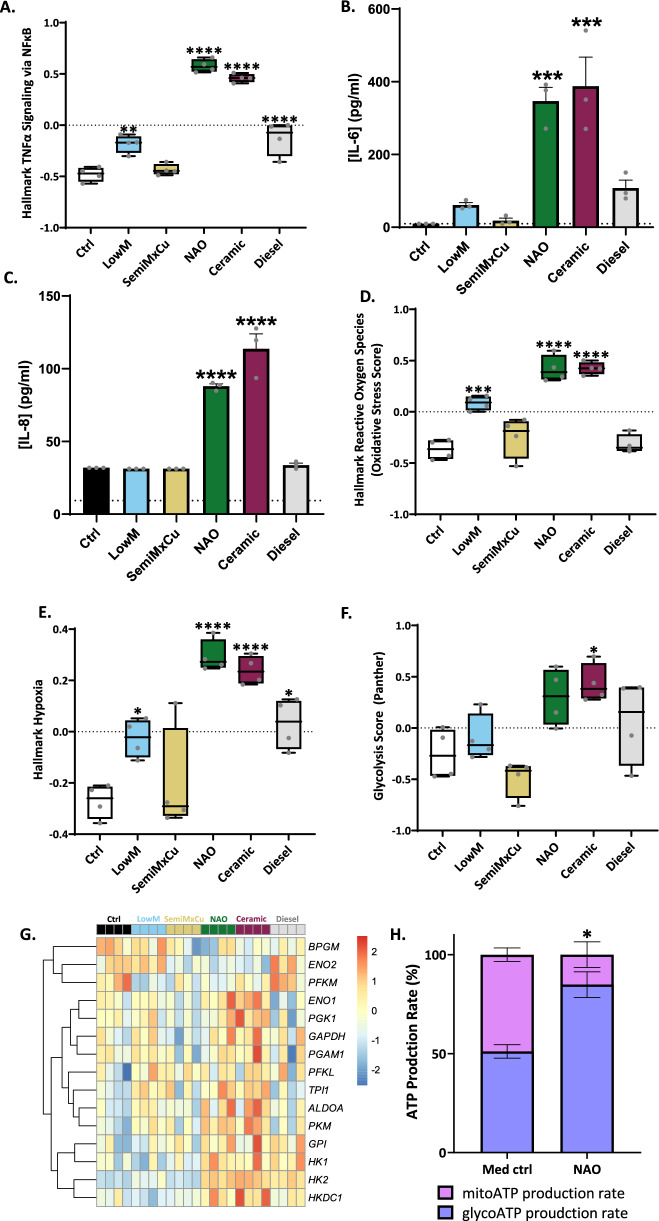
NAO- and ceramic-derived brake-wear PM strongly induce an inflammatory response and drive glycolytic reprogramming. ATII cells and primary ATII cells were exposed to 8 µg/cm^2^ of the 6 different PM types for 24 h, after which various markers of inflammation were examined. **A** GSVA for Hallmark TNFα signalling via NFκB **B** ATII cell IL-6 protein secretion was determined after exposure to 8 µg/cm^2^ of the 6 different PM types for 24 h. Determined via ELISA. **C** ATII cell IL-8 protein secretion was determined after exposure to 8 µg/cm^2^ of the 6 different PM types for 24 h. Determined via ELISA. **D** GSVA for Hallmark Reactive Oxygen Species (Oxidative Stress Score). **E** GSVA for Hallmark Hypoxia. **F** GSVA of Panther Glycolysis pathway. **G** Heatmap visualising the expression of genes within the GSVA of Panther Glycolysis pathway. **H** ATP Production Rate % from mitochondria, and from glycolysis in the medium control and NAO BWPM. Negative control represents cells that were not exposed to PM. In A: Box contains median, upper, and lower quartiles, with whiskers representing the range. In B, C, and H: Bars represent mean + SEM. In A-F: A RM one-way ANOVA test was used with a Dunnett’s post-hoc test. In H, the ‘*’ represents a significantly increased glycoATP production rate in NAO compared to med ctrl, determined using a two-tailed paired t-test. Statistically significant values are indicated with the star notation on the graphs. * = *p* ≤ 0.05, ** = *p* ≤ 0.01, *** = *p* ≤ 0.001, **** = *p* ≤ 0.0001

Gene Set Variation Analysis (GSVA) (Fig. 3D and Fig. S2A–E) identified similarities between the effects of NAO and ceramic brake-wear PM. Amongst the most upregulated Hallmark pathways caused by exposure to NAO or ceramic brake-wear PM was ‘Hallmark TNFα Signaling via NFκB’ and, notably, this effect was greater than that observed with other brake-wear PM or diesel PM (Fig. 4A). This potent proinflammatory effect was confirmed by measurement of cytokine release where only NAO and ceramic brake-wear PM induced a significant increase in interleukin-6 (IL-6) and interleukin-8 (IL-8) secretion (Fig. 4B, C respectively). A similar trend in responses was confirmed using primary ATII cells (Fig. S3A, B).

Other Hallmark pathways upregulated by NAO and ceramic brake-wear PM included ‘Hallmark Reactive Oxygen Species’ (Fig. 4D), and ‘Hallmark Hypoxia’ (Fig. 4E). Enrichment for ‘Hallmark Reactive Oxygen Species’ is consistent with our observation that NAO and ceramic brake-wear PM caused oxidative stress (Fig. 2). As the ‘Hallmark Hypoxia’ pathway is linked to a metabolic shift towards glycolysis, GSVA was used to interrogate glycolysis-associated genes using the Panther Glycolysis pathway (Fig. 4F). The Glycolysis Score was highest in response to NAO and ceramic brake-wear PM, driven by increased expression of a range of genes including *hexokinase-2* (*HK2*) and *pyruvate kinase M1/2* (*PKM*) (Fig. 4G). This shift in cellular metabolism was confirmed by Seahorse analysis which identified a significant increase in the proportion of ATP generation through glycolysis compared to oxidative phosphorylation in cells exposed to NAO brake-wear PM (Fig. 4H, and Fig. S3C).

**Fig. 5:**
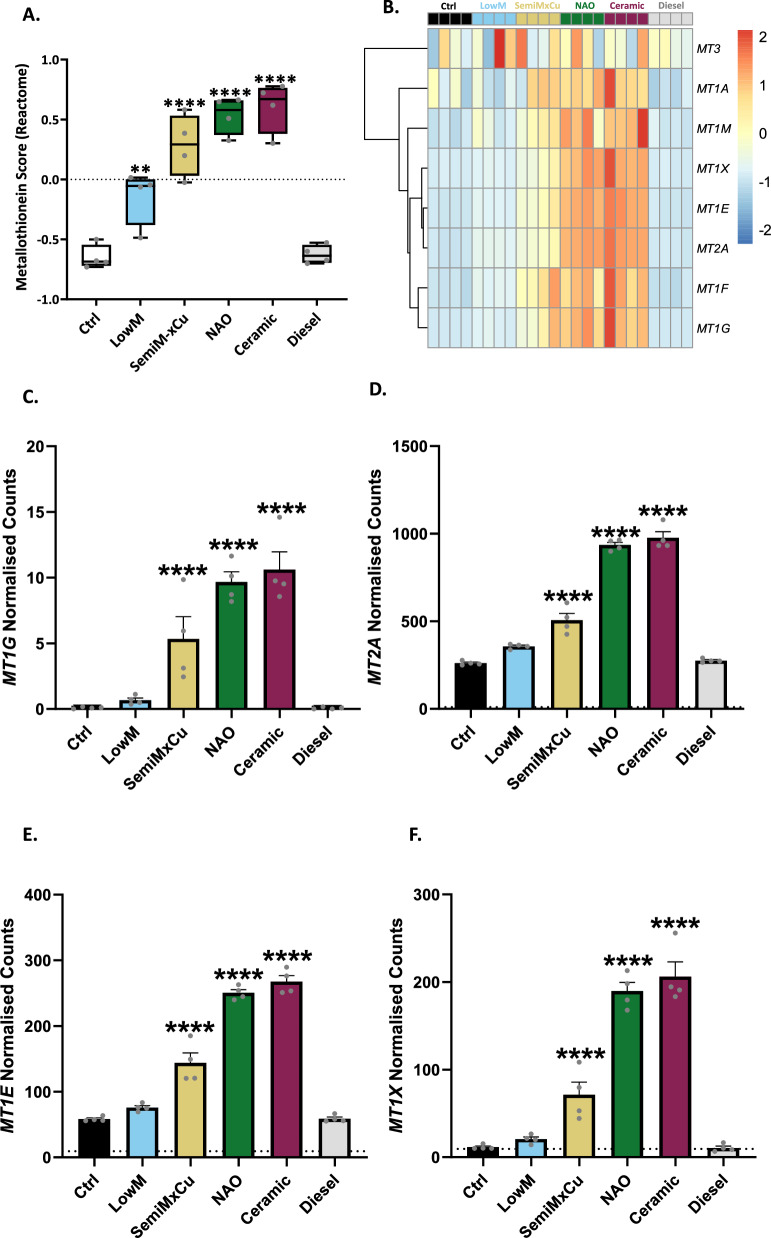
NAO- and ceramic-derived PM were most potent at perturbing metal ion homeostasis. ATII cells and primary ATII cells were exposed to 8 µg/cm^2^ of the 6 different PM types for 2–24 h, after which various markers of metal ion homeostasis were examined. **A** GSVA of “Metallothioneins bind metals (Reactome)” pathway. **B** Heatmap of genes within the Metallothioneins bind metals (Reactome)” pathway. **C** Normalised counts for *MT1G* (counts per million, determined with same RNA-Seq dataset as Fig. 3). **D** Normalised counts for *MT2A* C. Normalised counts for *MT1E*. D. Normalised counts for *MT1X*. In A: Box contains median, upper, and lower quartiles, with whiskers representing the range. A RM one-way ANOVA test was used with a Dunnett’s post-hoc test. In C-F: Bars represent mean + SEM of the normalised counts, significant differences compared to the ctrl were determined using a generalised linear model with edgeR. Statistically significant values are indicated with the star notation on the graphs. ** = *p* ≤ 0.01, **** = *p* ≤ 0.0001

Since metal content is a key difference between brake-wear PM and diesel PM, we also investigated the metal homeostasis responses to the different sources of vehicle-derived PM. Applying GSVA to examine the “Metallothioneins bind metals” Reactome pathway, which contains genes that encode members of the metallothionein family of proteins. We identified that all brake-wear PM types induced increased metallothionein score, with NAO and ceramic brake-wear PM causing the greatest upregulation, whilst no increase was observed in response to diesel PM (Fig. 5A, B). Furthermore, we identified a significant increase in metallothionein-encoding gene expression including *MT1G*, *MT2A*, *MT1E*, and *MT1X*, in response to SemiMxCu, NAO, and Ceramic brake-wear PM, with the magnitude increase being higher in response to the latter (Fig. 5C–F and Fig. S4A, B). This indicates that brake-wear PM can perturb cellular metal ion homeostasis, with NAO and ceramic brake-wear PM leading to the greatest effect.

In summary, this shows that brake-wear PM derived from NAO and ceramic brake pads have the greatest propensity to perturb ATII cell homeostasis. Importantly, across a range of measured endpoints, the effects of these PM were greater than diesel exhaust PM.

### Compositional analysis of vehicle-derived PM

**Fig. 6:**
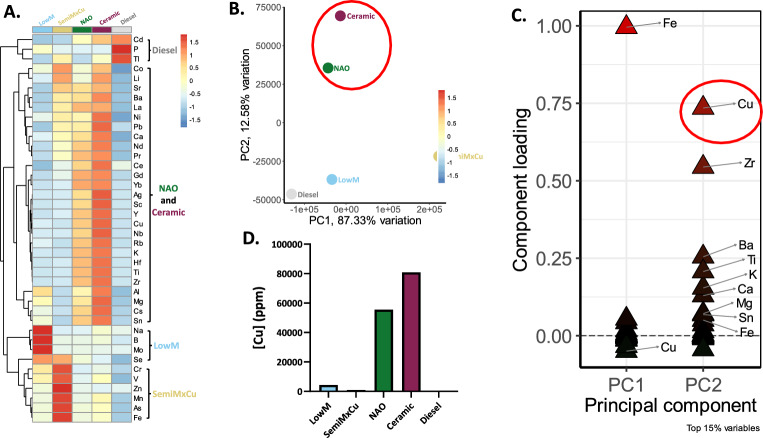
Different vehicle-derived PM types have distinct elemental characteristics, with NAO and ceramic BWPM differentiated by high copper content. 5 different vehicle-derived PM types were collected using a high-volume cascade impactor, after which, the whole particles were digested and analysed via ICP-MS. **A** Heatmap showing element concentrations with hierarchical clustering, to group elements enriched in each bulk PM type. **B** A biplot comparing the first two principal components for the bulk PM. NAO and Ceramic are separated along PC2 (circled in red). **C** A loadings plot showing the top 15% of elements that are contributing to the direction of the PCs for the bulk PM. For example, high iron concentrations are shifting PC1 values higher (as in the case of SemiMxCu), or high copper concentrations are shifting PC2 values higher (as in the case of NAO and ceramic). **D** Copper concentration in bulk PM demonstrating higher copper concentrations in NAO and ceramic brake-wear PM

Given our identification of type-specific brake-wear PM effects, we subsequently characterised the elemental composition of each PM type using Inductively Coupled Plasma Mass Spectrometry (ICP-MS) (Table S1). Hierarchical clustering identified that the different sources of vehicle-derived PM were elementally distinct (Fig. 6A). NAO and ceramic brake-wear PM had similar compositional profiles, defined by higher concentrations of certain metals, especially copper, zirconium, and titanium relative to the other tested PM types.

Given that NAO and ceramic brake-wear PM both exerted similar biological effects and have the most similar elemental composition, we wanted to identify what elements were most uniquely enriched in these particle types and could be responsible for driving these differential effects. Therefore, we analysed the compositional data using PCA. NAO and ceramic brake-wear PM were closely associated and distinct from the other PM types (Fig. 6B). Applying a loadings plot to determine the factors responsible for driving the principal components identified that the concentration of copper was most important for separating PM types (Fig. 6C). Consistent with this, copper concentrations were 16-fold higher in NAO and ceramic brake-wear PM than in LowM brake-wear PM (the next most copper-enriched source), 68-fold higher than SemiMxCu, and 300-fold higher than in diesel PM (Fig. 6D). Together these analyses identify that NAO and ceramic brake-wear PM are elementally distinct from other PM types and that the enrichment of copper is the key element differentiating PM from these brake pads from other brake-wear PM types.

### Copper-dependent effects of vehicle-derived PM

**Fig. 7:**
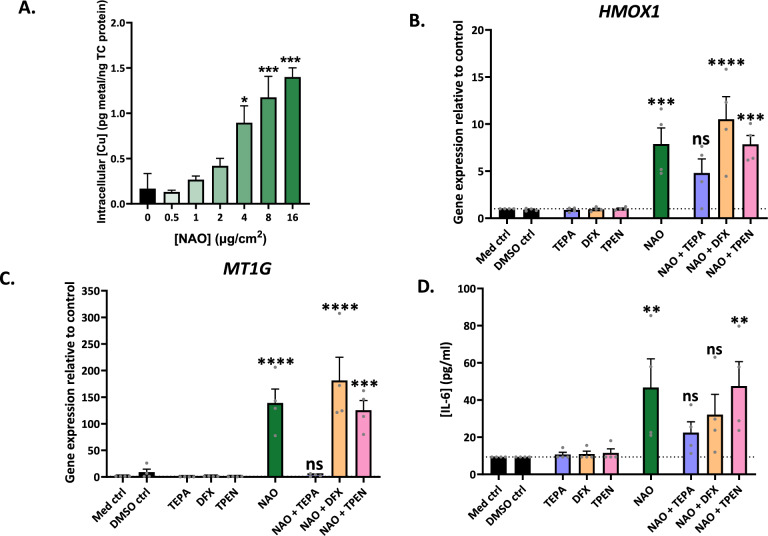
Copper drives the observed effects of NAO brake-wear PM. ATII cells were exposed to 6 concentrations of NAO BWPM (0.5, 1, 2, 4, 8, and 16 µg/cm^2^) for 24 h, after which the supernatant was harvested and the cells were washed 3 times before lysis with Chelex-100 treated MilliQ H2O. The lysates were then analysed for their elemental composition using ICP-MS, to determine the concentrations of the metals in cells after NAO BWPM exposure. **A** Intracellular copper concentration following NAO BWPM exposure. n = 3. Following this, ATII cells were exposed to either metal chelators alone (TEPA 50 µM, DFX 12.5 µM, or TPEN 1 µM), NAO BWPM (8 µg/cm^2^) alone, or the NAO BWPM and metal chelators together (NAO + TEPA, NAO + DFX, or NAO + TPEN) for 24 h. Following this, *HMOX1* gene expression (**B**), *MT1G* gene expression (**C**), and IL-6 protein secretion (**D**) were examined. n = 4. In all cases, bars represent mean + SEM, and a RM one-way ANOVA test was used with a Dunnett’s post-hoc test to test for significance. * = *p *≤ 0.05. ** = *p* ≤ 0.01, *** =* p* ≤ 0.001. **** = *p* ≤ 0.0001

We next investigated whether the type-specific brake-wear PM effects of NAO and ceramic brake pads we had identified were a consequence of copper enrichment. We focused on NAO brake-wear PM, given NAO brake pads are widely used. ATII cells were exposed to NAO brake-wear PM, and ICP-MS was performed to determine intracellular elemental composition. Copper accumulated intracellularly in a dose-dependent manner (Fig. 7A). Importantly, copper was the only element tested that increased in a dose-dependent manner that did not reach a peak at the highest studied concentration of NAO, potentially implying a lack of effective homeostatic control.

Having confirmed intracellular copper uptake, we then explored the consequences of exposure in the presence of different metal chelators: copper-selective tetraethylenepentamine (TEPA), iron-selective desferrioxamine (DFX), and zinc-selective N,N,N′,N′- *tetrakis*-(2-pyridylmethyl)ethylenediamine (TPEN). ATII cells were co-exposed to NAO brake-wear PM and the metal chelators. In the presence of copper-selective TEPA, NAO PM did not induce a significant increase in either the oxidative stress marker *HMOX1* (Fig. 7B), or the metal homeostasis marker *MT1G* (Fig. 7C). However, these responses were still observed in the presence of either iron-selective DFX, or zinc-selective TPEN. Furthermore, exposure to NAO brake-wear PM in the presence of copper-selective TEPA also resulted in the largest abrogation of pro-inflammatory IL-6 secretion (Fig. 7D). Together, these data suggest that copper is an important driver of the detrimental effects of NAO brake-wear PM on alveolar homeostasis.

### Pseudohypoxic HIF activation by vehicle-derived PM

**Fig. 8:**
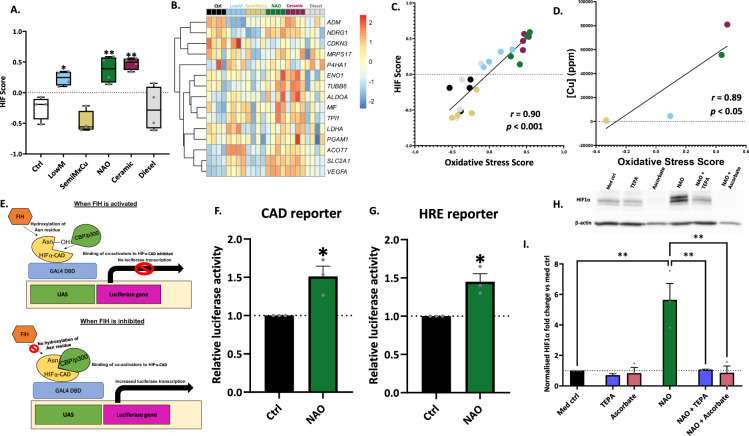
NAO BWPM induces pseudohypoxic HIF pathway activation in a copper-dependent manner. GSVA was used to interrogate a 15-gene signature indicative of HIF transcription factor signalling to generate a HIF score **A** GSVA of a 15-gene set HIF score. **B** Heatmap of genes within the HIF Score. **C** Correlation between the HIF score and Oxidative Stress Score. **D** Correlation between [Cu] and Oxidative Stress Score. **E**–**G**: ATII cells were transfected with either a hypoxia response element (HRE) reporter firefly luciferase plasmid, or hypoxia-inducible factor α C-activation domain (HIFα-CAD) firefly plasmid, and both with a *Renilla* luciferase control plasmid using lipofectamine LTX. Cells were then exposed to 8 µg/cm^2^ of NAO BWPM for 24 h and luciferase activity was determined using a Promega Dual-Luciferase^®^ Reporter Assay System. E. The mechanism of the HIF-CAD reporter assay. F. FIH inhibition following NAO BWPM exposure was determined using a HIFα-CAD reporter. n = 3. G. HRE binding following NAO BWPM exposure was determined using an HRE reporter. The plasmid contained an HRE binding region, and increased HIF transcription factor complex binding to this HRE binding site causes increased luciferase activity. PHD inhibitor DMOG was used as a positive control. n = 3. H and I: ATII cells were exposed to NAO brake-wear PM (8 µg/cm^2^), TEPA (50 µM), NAC (10 mM), or Ascorbate (5 mM) alone for 24 h. The cells were also exposed to NAO and TEPA together for 24 h, with no pre-treatment, NAO + NAC and NAO + Ascorbate refer to 24 h NAO exposure after a one-hour pre-treatment with respective agents. H. Representative HIF1α western blot showing the impact of NAO BWPM on HIF1α stabilisation, as well as the impact of copper chelator TEPA. I. Densitometric analysis of the western blots. n = 3. In F, G, and I data was represented as mean + SEM. In A: Box contains median, upper, and lower quartiles, with whiskers representing the range. In A and I, a RM one-way ANOVA test was used with a Dunnett’s post-hoc test. In C and D, a Pearson’s correlation was used. In F and G, and two-tailed paired t-test was used

We and others have previously identified that oxidative stress acting through inhibition of Factor Inhibiting HIF (FIH) can activate the key hypoxia response transcription factor, hypoxia-inducible factor (HIF) in an oxygen-independent manner, a process termed pseudohypoxic HIF activation [25–27]. Within the lung, this has been proposed to promote progressive fibrosis [27]. Since our transcriptomic analyses identified that NAO brake-wear PM can induce oxidative stress (Fig. 2B–D) as well as increase the expression of genes associated with hypoxia (Fig. 4E), we investigated whether copper-rich brake-wear PM promoted pseudohypoxic HIF activation. To test if HIF signalling was occurring in response to PM, GSVA was conducted on the RNA-Seq dataset to determine the HIF score, using a validated 15-gene signature indicative of HIF signalling [28]. NAO and ceramic brake-wear PM exposure resulted in a significantly increased HIF score (Fig. 8A), with increased expression of genes including *vascular endothelial growth factor A* (*VEGFA*) and *solute carrier family 2 member 1* (*SLC2A1*) (Fig. 8B). The HIF score showed a strong positive correlation with the oxidative stress score, suggesting that the HIF signalling could be driven by oxidative stress (r = 0.90, *p* < 0.001; Fig. 8C). Furthermore, we identified a strong positive correlation between the concentration of copper within the different PM types, and their oxidative stress score (r = 0.89, *p* < 0.05; Fig. 8D).

To investigate if FIH activity was altered in response to NAO brake-wear PM, a GAL4 DNA-binding domain upstream activation sequence (DBD-UAS) reporter system was used: HIF1α-CAD is bound to the GAL4 DBD, which binds to an UAS [27, 29]. When FIH is inhibited, luciferase activity is higher (Fig. 8E). Exposure to NAO brake-wear PM induced a significant increase in luciferase activity, consistent with FIH inhibition (Fig. 8F). Next, we used a hypoxia-response element (HRE) reporter assay to determine if HIF1α is subsequently able to drive signalling via hypoxia-response elements (HREs), thereby altering the expression of hypoxia-inducible genes. With this system, increased activation of signalling through HIF1α-HRE binding results in increased luciferase activity. Exposure of ATII cells to NAO brake-wear PM induced a significant increase in luciferase activity, indicating NAO-induced increased binding of HIF transcription factor complexes to HREs (Fig. 8G). Lastly, protein concentrations of the key inducible sub-unit of HIF transcription factor complex HIF1α were assessed. Exposure of ATII cells to NAO brake-wear PM resulted in a significant increase in HIF1α stabilisation (Fig. 8H, I). This response was diminished by a pre-treatment with the antioxidant ascorbate, and abrogated by co-treatment with copper-selective chelator TEPA (Fig. 8H, I). Together these findings are consistent with copper-enriched PM promoting pseudohypoxic HIF pathway activation via copper-induced oxidative stress and inactivation of FIH. * = *p* ≤ 0.05. ** = *p* ≤ 0.01, *** =* p* ≤ 0.001. **** = *p* ≤ 0.0001.

## Discussion

A major source of PM in urban environments is road traffic, but much of our understanding of the toxicity of traffic-related PM relates to diesel exhaust emissions. However, non-exhaust emissions, especially from brake wear, represent an increasing component of traffic-related PM, about which there is a paucity of information. Here, we have shown that compared to diesel exhaust PM, brake-wear PM was more potent in perturbing cellular homeostasis in a manner dependent on PM copper content, associated with NAO and ceramic brake pads. The alterations in biological pathways observed here have been linked to a range of diseases including chronic obstructive pulmonary disease (COPD), idiopathic pulmonary fibrosis (IPF), cancer, cardiovascular diseases, Alzheimer’s disease, and premature ageing [23, 24, 30–33]. However, non-exhaust emissions are only recently being considered for regulation, and these proposals do not include compositional regulation for the protection of human health [34].

Oxidative stress is one of the key established mediators of fine PM toxicity and has been associated with the pathogenesis of multiple diseases [30, 35, 36]. PM has been shown to promote the generation of ROS through various mechanisms, which can offset the oxidant-antioxidant balance [37]. The endpoint of this is oxidative damage to cell membranes, proteins, and DNA leading to downstream consequences such as inflammation and cell death [38–40]. We demonstrated here that on a mass basis, NAO and ceramic-derived brake-wear PM induced the greatest generation of ROS, as well as the highest magnitude increase in the expression of oxidative stress-associated genes compared to the other brake-wear PM types, and diesel PM. Other groups have identified that brake-wear PM can induce ROS generation and antioxidant responses in vitro [41–43]. However, we elaborate on this by identifying the response to these PM across the whole transcriptome. Furthermore, we demonstrate that these responses are brake-pad specific, and are more potent than diesel PM.

Downstream of oxidative stress, inflammation has been identified as a key driver of PM2.5 cellular toxicity. The alveolar epithelium is one of the initial sites of fine PM deposition, and this can result in localised inflammation which has the potential to perturb epithelial barrier function [44]. It has also been suggested that PM exposure can drive systemic inflammation leading to multi-organ toxicity. For example, pro-inflammatory mediators may enter the circulation and act directly on the heart leading to cardiac remodelling and disease development [45]. It has been shown that those presenting with myocardial infarction were more likely to have been exposed to road traffic emissions in the previous hours [46]. Using the RNA-Seq dataset generated in this study, we identified that the greatest magnitude increase in the expression of pro-inflammatory genes was seen in response to NAO and ceramic brake-wear PM. Furthermore, we demonstrated that only NAO and ceramic brake-wear PM could stimulate the release of pro-inflammatory mediators (IL-6, IL-8) following exposure of ATII cells to non-cytotoxic concentrations of PM. Previous research has demonstrated that exposure of U937 monocyte-derived macrophages to mixed brake-abrasion dust collected from a ventilation duct at a testing plant induces IL-8 release, as well as reduced macrophage phagocytic capacity [47]. Moreover, our findings align with a previous murine in vivo study that demonstrated pro-inflammatory mediator release in response to brake-wear PM, however, the underlying mechanism remained uncertain [48]. Here, we show that copper is a key determinant of these responses.

The main difference in elemental composition between diesel PM and brake-wear PM measured here is the enrichment of metals in the latter. Consistent with this difference, we demonstrated that all brake-wear PM types could upregulate the expression of genes encoding metallothioneins, cysteine-rich metal-chelating proteins [49]. Metallothioneins are canonically described as zinc-binding proteins [50], which play an important role in regulating the intracellular free concentration of zinc [49]. When intracellular free zinc concentrations increase, zinc binds to metal regulatory transcription factor 1 (MTF1), promoting nuclear translocation and binding to promoter sequences on metallothionein genes, increasing their expression to maintain zinc homeostasis [49]. In this context, it is notable that SemiMxCu-derived brake-wear PM contained more zinc than any of the other vehicle-derived PM types examined, however, NAO and ceramic brake-wear PM still induced the highest magnitude response in metallothionein gene expression. Notably, metallothioneins also bind copper in vivo, and have a well-described role in the detoxification of cadmium [51, 52]. Here, we demonstrated that the metallothionein induction by NAO brake-wear PM was driven by copper, consistent with the evidence that metallothionein proteins have a higher affinity for copper than zinc [49, 53]. While we have previously shown exposure to metal-rich PM from an underground railway station can cause upregulation of metallothioneins in bronchial epithelial cells [54], to our knowledge, this is the first report that brake-wear PM can perturb zinc homeostasis and induce metallothionein expression.

Exposure to PM, copper, and oxidative stress have independently been associated with the pathogenesis of chronic lung diseases [30, 55–58]. However, the mechanisms of how PM and copper might drive these are poorly understood. Recent research has demonstrated that oxidative stress in fibroblasts can induce HIF pathway activation under normal oxygen conditions due to inhibition of Factor Inhibiting HIF (FIH) [27]. This pseudohypoxic HIF activation underlies an increase in ‘bone-type’ (pyridinoline) collagen crosslinking which is characteristic of fibrosis and tissue stiffening—a key mechanistic driver of various lung diseases [25, 27]. Moreover, pseudohypoxic HIF activation has been identified as a pathogenic mechanism in several cancers. There is also a growing interest in understanding how PM2.5 may drive lung cancer in never-smokers through non-mutagenic mechanisms [25, 59]. Given that NAO and ceramic brake-wear PM induced oxidative stress, and hypoxia response genes were shown to change in our RNA-Seq data, we investigated if such vehicle-derived sources of PM could inactivate FIH. Consistent with this, the transcriptome of NAO brake-wear PM-exposed ATII cells exhibited an increased HIF score and this correlated with the oxidative stress score. A causal link was further evidenced by the finding that NAO brake-wear PM could stabilise HIF1α protein (the key inducible sub-unit of the HIF complex) in an oxidative stress- and copper-dependent manner. Finally, we demonstrated that NAO brake-wear PM caused a significant inhibition of FIH. Together these data demonstrate that NAO brake-wear PM can induce HIF signalling in an oxidative stress/copper-dependent fashion. These findings have significant implications for understanding how specific forms of non-exhaust PM, especially those containing high concentrations of copper, may contribute to the development and progression of many chronic lung diseases.

Brake wear and tyre wear are the leading sources of atmospheric copper, contributing to 47% of airborne concentrations according to the UK emissions inventory [9]. Copper is often used as a tracer for brake-wear PM in epidemiological studies investigating the impact of non-exhaust emissions, with various short-term and long-term studies finding an association between PM copper and increases in morbidity/mortality [56, 60–66]. Results from the Atlanta Commuters Exposures study observed acute decreases in lung function in a cohort of young adults which were positively associated with PM copper exposure during commuting, with no other metals correlating with lung function changes [63]. Similarly, a retrospective panel study found that higher concentrations of copper within PM2.5 were associated with acute decreases in lung function in COPD patients [56]. In support of this, we demonstrate here that NAO PM-derived copper accumulates intracellularly following exposure, and chelation of NAO PM-derived copper abrogates a range of endpoints associated with toxicity, providing a potential mechanistic explanation of these epidemiological findings. Interestingly, Selley et al. conducted a similar experiment but with monocyte-derived macrophages and mixed brake-abrasion dust collected from a ventilation duct at a testing plant, as mentioned above [47]. They however did not observe an increase in intracellular copper in a concentration-dependent manner in response to these PM. This could be due to the difference in braking materials used within the plant, or simply due to differences in uptake between monocyte-derived macrophages, and the alveolar epithelial cells used here [47]. Figliuzzi et al. exposed A549 cells to brake-wear PM derived from 4 different unspecified brake pad types. They suggested that there was an association between brake-wear copper concentration and effect, but their study used a concentration of 100 µg/ml for several of these experiments (four times higher than used in our work), and this caused marked cytotoxicity [42]. Moreover, where they suggest a concentration–response effect, there is no investigation of causality. Our findings elaborate on this by directly ascribing observed effects to the copper within the brake wear.

Legislation changes relating to non-exhaust PM in the interest of public health have not yet been implemented. However, the toxic effects on aquatic life of copper from brake-wear PM washed from roads to waterways has resulted in some US states (including California and Washington) introducing laws that restrict copper usage within brake pad formulations [67]. This has in turn incentivised the development of novel brake pad types, such as the SemiMxCu brake pads examined here. Currently, there is no legislation governing non-exhaust emissions in Europe, although the proposed Euro 7 standards aim to implement changes [34] with current plans aiming to set brake-wear PM_10_ emissions limits to 7 mg/km/vehicle, with an aim to eventually reach 3 mg/km/vehicle [34]. However, our research suggests that legislation changes specifically to reduce copper content within brake pads could also be beneficial to public health. While it is important to note that currently available copper-limited brake pads have been known to have a higher wear rate, recent advances in tribological technology have found copper-limited brake pads that utilise stainless steel particles appear to perform better than copper-containing pads, with lower wear rates [68]. Widespread implementation of legislation to reduce brake pad copper content would likely further drive tribological research into developing better friction materials with reduced wear rates without the need for copper, which would lower overall exposure to toxic brake-wear PM emissions.

## Limitations of the study

Given that our focus was on metals within the PM, other potentially toxic components were not considered. For example, brake-wear PM contains over 150 different organic compounds [18]. Furthermore, given that only 1% of the elemental composition of diesel PM was accounted for in the analysis here, carbonaceous compounds are likely to contribute to much of the unaccounted mass. Indeed, studies have shown that diesel PM contains high concentrations of organic compounds, including mutagenic polycyclic aromatic hydrocarbons (PAHs) [69, 70].

Brake-wear PM toxicity was assessed using 2D monocultures. Future studies could confirm these findings using a more complex 3D multi-cell co-culture that better represents particle inhalation by modelling apicobasal polarity, more realistic spatial organisation, and allowing for cell–cell interactions [71]. Lastly, the concentration of PM (8 µg/cm^2^) used is likely higher than in vivo exposures. However, as airway lining fluid in vivo is only ~ 0.05 µm thick, while the in vitro exposure thickness here was ~ 3 mm, this could have implications for the effects of the dissolved compounds [72]. Importantly, this concentration is in line with, and often lower than concentrations used for similar previous in vitro studies [42, 43, 47, 73, 74].

## Conclusions

In summary, we have demonstrated that copper is a key determinant of the ability of brake-wear PM to perturb alveolar epithelial cellular homeostasis. Importantly, copper-enriched brake-wear PM has a greater detrimental effect than diesel exhaust PM. The perturbations caused by copper-enriched PM have the potential to contribute to the development and progression of a range of diseases, negatively impacting healthy living and lifespan. Our results suggest that current legislation, which focuses on PM exhaust emissions, may be inadequate to mitigate the health effects of vehicle-derived PM, and that regulations also need to target not only non-exhaust PM emissions, but also specific components of PM.

## Methods

### PM sampling

PM2.5–0.1 (aerodynamic diameter 2.5–0.1 µm) was collected onto a polyurethane foam collection substrate using a high-volume cascade impactor (HVCI) operating at 0.9 m^3^ air inflow per minute. PM was extracted from foam filters by sonication into methanol and dried under nitrogen gas. Brake-wear PM was generated from ECE low-metallic brake pads (LowM), semi-metallic brake pads with limited copper (SemiMxCu), non-asbestos organic brake pads (NAO), and ECE-NAO hybrid brake pads (Ceramic). Diesel exhaust PM was also collected. Specific methodological differences in the collection of the various PM types are described in detail in the subsequent sections.

### Brake-wear PM

Brake-wear PM (PM2.5–0.1) from four different commonly used brake pad types was collected under experimental conditions at the Technical University of Ilmenau (Ilmenau, Germany) [48, 75]. The HVCI was placed in a large, air-conditioned metal box containing a wheel and brake assembly rotating freely during braking motions. Air supply was reduced during particle collection to reduce particle losses. Each run contained a set sequence of braking actions to replicate city, motorway, and country road driving conditions. This was achieved by braking from different running speeds (sometimes at low speeds, sometimes to a full stop), temperatures, and forces [48, 75].

### Diesel PM collection

Diesel PM was collected from a heavy-duty 12.6 L incline 6-cylinder engine on a test bench (Euro III standard-compliant combustion settings) at Eindhoven University of Technology, Netherlands [76]. The engine was fuelled with commercial diesel (EN590) and ran at 1200 RPM to mimic typical motorway conditions [76].

### Preparation of PM samples

PM samples were stored as a dry powder, before being resuspended in 18.2 MΩ·cm ultrapure water to 2 mg/ml, with vortexing for one minute and sonication in an iced bath sonicator for 15 min. Resuspended PM samples were then aliquoted and stored at − 20 °C until use [76].

### ATII cell culture

An immortalised alveolar type-II (ATII) cell line was used in this study as previously reported [77–80]. These cells have been demonstrated to express markers of ATII cells, including surfactant protein C [79].

ATII cells [77–81] were maintained in culture in either T25 or T75 tissue culture flasks in DCCM1 (Biological Industries, Beit HaEmek, Israel) supplemented with 10% foetal bovine serum (FBS), 50 units/ml penicillin, 50 µg/ml streptomycin, and 2 mM L-Glutamine (all from Gibco, ThermoFisher, Massachusetts, USA) (hereafter referred to as complete DCCM1), and passaged twice a week using trypsin/EDTA digestion, seeding at a density of 100,000 or 300,000 cells in T25 and T75 flasks, respectively. Cells were incubated at 37 °C, 5% CO_2_. For exposure, ATII cells were seeded at a density of 30,000 cells in 600 µl complete DCCM1 in Nunc 24 well plates (ThermoFisher) and grown until 80–90% confluent, whereupon cells were serum starved overnight (complete DCCM1 without FBS, hereafter referred to as serum starvation DCCM1).

### Challenge of cells with PM

Following serum starvation, cells were exposed to 4–32 µg/cm^2^ (equivalent to 12.5–100 µg/ml) of PM, prepared as above, and diluted into starvation DCCM1 for 2–24 h. In some experiments, metal chelators (TEPA 50 µM, DFX 12.5 µM, or TPEN 1 µM; all from Sigma) were added to the PM challenge. Following exposure, supernatants were harvested and centrifuged at 16,000 × *g* 4 °C for 10 min, to pellet debris. Clean supernatants were stored in Eppendorf tubes at − 80 °C until use.

### Primary ATII cells

Primary ATII cells were extracted from macroscopically normal human lung tissue deemed surplus to clinical diagnostic requirements, obtained following video assistant thoracoscopic surgery (VATS). Ethical approval was gained from the Southampton and South West Hampshire and the Mid and South Buckinghamshire Local Research Ethics Committees, and all subjects gave written informed consent. The isolation of primary ATII cells was performed as previously published [82, 83].

Prior to use, primary ATII cells were seeded in 48-well plates at 172,000 cells per well in a volume of 400 µl of DCCM-1 medium supplemented with 10% newborn calf serum (NCS), penicillin (100 units/ml), streptomycin (100 µg/ml), and L-Glutamine (2 mM) (PSG) (all from Invitrogen, Massachusetts, USA). After 48 h cells were gently washed to remove non-adherent cells, and the medium was replaced with DCCM1 medium supplemented with 10% NCS and 1% PSG, this process was repeated every 48 h until day 6. After 6 days, the medium was replaced with starvation medium (DCCM1 medium supplemented with 1% PSG, 0% NCS). 24 h later, cultures were exposed to 8 µg/cm^2^ (25 µg/ml) PM2.5–0.1 suspended in the starvation medium for 24 h (350 µl exposure volume). Supernatants were then harvested and processed as above.

### LDH assay

Cytotoxicity of the PM exposures was assessed using a CytoTox 96^®^ Non-Radioactive Cytotoxicity Assay Kit (Promega, Wisconsin, USA). A representation of total cellular LDH was obtained by culturing a well of cells treated as a PM-free control, washed with PBS, and then lysed with 600 μl 1% Triton-X-100 in starvation medium. This lysate was then processed (centrifugation, storage) as described for the supernatants above. LDH assay was performed as per the manufacturer’s instructions, and an LDH standard curve was constructed using 1 in 2 serial dilutions of the total LDH lysate. Absorbance was measured at 492 nm using a MultiSkan FC spectrophotometric plate reader (ThermoFisher), and LDH release was calculated as a percentage of total cellular LDH using the standard curve. All samples were tested in duplicate.

### ***2***′***, 7***′***-Dichlorodihydrofluoroscein diacetate (H2-DCF-DA) assay***

To determine ROS generation in response to PM exposures, the ROS-sensitive dye 2′, 7′-Dichlorodihydrofluoroscin diacetate (H_2_-DCF-DA; Sigma, New Jersey, USA) was used. ATII cells were seeded in 96-well plates at 5000 per well in 100 µl of complete DCCM1, after 24 h, the cells were serum-starved for 16 h. For the assay, cells were washed twice with 150 µl of phenol red-free HBSS supplemented with Ca^2+^ and Mg^2+^ (ThermoFisher), before being loaded with H_2_-DCF-DA (40 µM in starvation DCCM1, 100 μl per well) for 45 min. Residual H_2_-DCF-DA dye was removed, cells were washed twice with phenol red-free HBSS supplemented with Ca^2+^ and Mg^2+^, and cells were exposed to PM as above. Fluorescence was measured using a Fluoroskan Ascent FL plate reader (Labsystems, Vantaa, Finland) (λ_exc_ = 485 nm, λ_em_ = 530 nm) after 24 h. 100 µM tert-Butyl hydroperoxide (TBHP; Sigma) was used as a positive control. All samples were tested in duplicate.

### RNA extraction and reverse transcription

Following PM exposure, cells were washed with 1 ml sterile phosphate-buffered saline (PBS) and lysed with 300 µl RNA lysis buffer (Monarch^®^ Total RNA Miniprep Kit, New England BioLabs, Massachusetts, USA) for 10 min to completely lyse cells. Lysates were stored at − 80 °C until further use.

RNA was extracted using a Monarch^®^ Total RNA Miniprep Kit (New England BioLabs) following the manufacturer's protocol, using the optional on-column DNase I digest, and eluting in 50 µl of 18.2 MΩ^.^cm ultrapure water. RNA quantity and purity were assayed using a NanoDrop™ spectrophotometer (ThermoFisher). 260/280 absorbance values of > 1.8 were considered of sufficient quality for downstream analyses. Total RNA samples were reverse transcribed to cDNA using a High-Capacity cDNA Reverse-Transcription Kit (ThermoFisher) following the manufacturer’s instructions (20 µl reaction volume) with a starting RNA concentration between 10 and 20 ng/µl of RNA.

### RT-qPCR

RT-qPCR was performed using TaqMan RT-qPCR gene expression assays (ThermoFisher), with *ATP5B* and *TOP1* used as housekeeping genes, the primers used in this study are described in Table 1. Reaction mixtures were prepared using 5 μl TaqMan Fast Advanced MasterMix, 2 μl ultrapure water, and 0.5 μl cDNA. Reactions were run using a Bio-Rad CFX96 Real-Time thermal cycler, with data analysed using Bio-Rad CFX Manager Version 3.1 (Bio-Rad, California, USA). Relative fold change was calculated using the 2^−ΔΔCt^ method [84], using a geometric mean of the housekeeping gene Ct values. All samples were tested in duplicate.

**Table 1:** Primers used in this study

Gene	Dye	Hs code
*ATP5B*	FAM	Hs00969569_m1
*GCLM*	FAM	Hs00978072_m1
*HMOX1*	FAM	Hs01110250_m1
*MT1G*	FAM	Hs04401199_s1
*MT2A*	FAM	Hs02379661_g1
*TOP1*	VIC	Hs00243257_m1

### RNA-Seq and data processing

Following PM exposure, RNA was harvested and extracted as described above, and samples were analysed by mRNA-Seq using Illumina Novaseq 6000 paired-end sequencing with 150-nucleotide read length and 20 million reads per sample (Novogene, Cambridge, UK). RNA library preparation was conducted using Novogene’s NGS RNA Library Prep Set (PT042). FASTQ raw reads were pseudoaligned to the human transcriptome (GRCh38) and quantified using kallisto [85]. The resulting data were imported into R using the Bioconductor tximport package, producing gene-level estimated counts which were used downstream with edgeR for differential expression analysis [86]. Data were filtered to remove lowly expressed genes using the edgeR filterByExp function which keeps genes with > 10 counts in at least 70% of samples [86]. Data were normalised with the Trimmed Mean of M-values (TMM) method, and relative gene expression compared to the control was then determined using a generalised linear model (glmQLFit), once again using edgeR [86]. Genes with a Benjamini–Hochberg false discovery rate (FDR) adjusted p-value of ≤ 0.05 were considered differentially expressed compared to the control. PCA was used to visualise variation within the dataset. Log_2_ counts-per-million (cpm) values were inputted into the PCA algorithm within the base R programming language (prcomp). The responses of genes within the Hallmark pathways were analysed using Gene Set Variation Analysis (GSVA), with log_2_ cpm values used as an input for the GSVA tool in R; groups were compared using a repeated-measures one-way ANOVA with a Dunnett’s post-hoc test with *p* ≤ 0.05 as the significance threshold [87]. Heatmaps were generated using the pheatmap package in R, with hierarchical clustering using Euclidean distance as the clustering metric and complete clustering.

### Pro-inflammatory mediator release

Pro-inflammatory mediator release was assessed using DuoSet^®^ IL-6 and IL-8 ELISA kits (R&D systems, Minnesota, USA), following the manufacturer’s instructions. All samples were tested in duplicate.

### Seahorse ATP-rate assay

Cells were prepared for the Seahorse ATP-rate assay as per the manufacturer’s guidance. Briefly, ATII cells were seeded at 2500 cells in 100 µl of DCCM1 complete medium per well in the central 6 wells of a Seahorse XF HS 8 well cell culture miniplate (Agilent Technologies, California, USA), and the next day serum-starved using DCCM1 starvation medium. On day 3, cells were challenged with 25 µg/ml PM (19 µg/cm^2^) in 80 µl DCCM1 starvation medium for 24 h, following which supernatants were removed, cells were washed with Seahorse medium (Seahorse DMEM + 10 mM glucose + 1 mM pyruvate + 2 mM glutamine; Agilent Technologies), and replenished with 80 µl Seahorse medium containing 25 μg/ml (~ 19 µg/cm^2^) PM, with incubation for 1 h at 37 °C without supplementation with CO_2_ and no air circulation. After 1 h, cells were washed, the PM-containing medium was replenished, and an ATP rate assay was conducted according to the manufacturer’s instructions using a Seahorse XF mini real-time metabolic analyser (Agilent Technologies, California, USA). At the end of the assay, supernatants were removed and cells were washed three times with PBS, before the addition of 20 µl of 5 × Laemmli lysis buffer. Lysate protein concentration was determined using a Pierce Micro BCA™ Protein Assay Kit (ThermoFisher). Data analysis was conducted using Agilent’s Seahorse analytics software, using total protein content for normalisation of the assay results. All samples were tested in duplicate.

### PM sample preparation for ICP-MS – bulk fraction

PM2.5–0.1 sample suspensions were thawed, vortexed briefly to resuspend, and then sonicated in an iced water bath sonicator for 10 min. 5 mg of PM was then acid digested in 5 ml aqua regia (1:3 12.6 M HNO_3_: 15.5 M HCl prepared from sub-boiled concentrated acids) and ~ 0.25 ml 27M HF (all acids from ROMIL-SpA™ concentrated acids; Romil, Cambridge, UK) in a CEM MARS6 microwave digestion system (CEM Corporation, North Carolina, USA) ramping to 200 °C in 15 min and with a 15 min hold time in 20 ml PFA MARSXpress vessels (CEM Corporation). Solutions were then evaporated to dryness on a hot plate at 130 °C and further digested overnight in ~ 5 ml 3% HNO_3_ at room temperature, followed by a final evaporation and then dilution with 5 ml 3% HNO_3_ spiked with In, Re, and Be as internal standards. Elemental concentrations were analysed using an Agilent 8900 QQQ ICP-MS (Agilent, USA), calibrated with multi-elemental standards prepared from single-element ICP-MS standards (Inorganic Ventures, Virginia, USA). Digest blanks to assess background signal were prepared using 18.2 MΩ·cm ultrapure water instead of PM2.5–0.1. The lower limit of detection for each element was calculated as the mean of the digest blanks plus three times the standard deviation.

### ICP-MS data analysis

Firstly, elemental data was represented as a heatmap generated using the pheatmap package in R, with hierarchical clustering with Euclidean distance and complete clustering used to identify elemental signatures. Following this, the dimensionality reduction technique principal component analysis (PCA) was used to visualise PM compositional differences. Bulk particle concentrations (ppm) were inputted into the PCA algorithm within the base R programming language (prcomp). Results were visualised using ggplot2 [88].

### Cell lysate sample preparation for ICP-MS

To determine the concentration of PM elements accumulating within cells following PM exposure, ATII cells were exposed to NAO brake-wear PM (0.5, 1, 2, 4, 8, or 16 µg/cm^2^) for 24 h, after which the supernatant was harvested and the cells were washed 3 times with PBS before lysis with 100 µl of Chelex^®^−100 treated MilliQ H_2_O (1.5 g of Chelex^®^−100 resin; Sigma added to 50 ml mΩ cm ultrapure H_2_O), agitated overnight at 4 °C, and then separated by centrifugation at 16,000 × *g* for 10 min and filtered through a 0.2 µm pore filter. 10 µl of the lysate was used to determine the cellular protein concentration within the cells using a Pierce Micro BCA™ Protein Assay Kit for normalisation of cellular elemental concentrations. 30 µl of the lysates were added to Teflon pots with 250 μl aqua regia and digested for 24 h at room temperature, followed by evaporation at 130 °C and the addition of 2 ml 3% HNO_3_ spiked with In, Re, and Be. The concentration of 19 different elements (As, Ba, Cd, Cu, Fe, Hf, La, Mg, Mo, Na, Nb, Nd, Ni, Sn, Sr, Ti, Yb, Zn, Zr) were determined using ICP-MS as above, and normalised to total cellular protein concentrations, measured in 10 μl lysate using a Micro BCA™ Protein Assay Kit.

### Cell exposures and lysis of protein for western blotting

After the PM exposure, cells were washed twice in ice-cold PBS, before lysis with 50 µl of 2 × Laemmli SDS sample buffer supplemented with 20 µl/ml cOmplete™ protease inhibitor (Roche, Basel, Switzerland) and 100 µl/ml PhosSTOP (Roche). Specific prolyl hydroxylase domain-containing 2 (PHD2) inhibitor IOX2 (250 µm) was used as a positive control for activation of HIF signalling. The lysates were stored at −80 °C until use.

### SDS-PAGE and western blotting

Lysates were probe sonicated with a Soniprep 150 machine (MSE supplies, Arizona, USA) for 3 × 10 s (5 µm amplitude) before total protein concentration was determined using a Pierce Micro BCA™ Protein Assay Kit (ThermoFisher). Lysates were then diluted in a sample loading buffer with 12.5% of β-mercaptoethanol as a reducing agent, to contain 20 µg of protein in a 30 µl final volume. Samples were heated to 95 °C for 5 min, and centrifuged at 16,000 × *g* for 5 min. For SDS-PAGE, polyacrylamide stacking and separation gels (10%) were prepared using 1 mm BioRad mini-PROTEAN^®^ hand-cast systems (Bio-Rad, Hercules, California, USA). Following this, 30 µl of each protein lysate sample was run by PAGE at a constant 100V with 0.3A limit for ~ 2 h or until the dye front reached the bottom of the gel. 7 µl of protein ladder (Precision Plus Protein Kaleidoscope; Bio-Rad, Hercules, California, USA) was run in the first lane of each gel. Following immersion in cold transfer buffer to remove salts and pre-shrink the gels, proteins were transferred to PVDF membranes at a constant 100V with a 0.3A limit for 2 h. Following transfer, PVDF membranes were blocked in 5% milk powder in TBS-T for 1 h. Membranes were probed for 24 h using mouse anti-human HIF1α antibody (1:1000, BD Biosciences, Wokingham, UK), or mouse anti-human β-actin antibody as a housekeeping protein (1:50,000, Merck, New Jersey, USA), followed by HRP-conjugated rabbit anti-mouse secondary antibody (1:1000, Cell Signaling Technology, USA). Proteins were visualised using Clarity Western Blotting ECL Substrate (Bio-Rad Laboratories, Watford, UK) using an Amersham Imager 600 (General Electric, Boston, USA). Details of the buffers used and gel recipes are included in the supplement.

Band densitometry was performed using ImageJ software on the raw blot images [89], with proteins of interest normalised to the housekeeping protein to account for inter-lane loading differences, and then compared to the medium control to derive a fold-change in the protein of interest.

### Reporter assays

HRE binding and FIH activity were assessed using luciferase reporter assays. ATII cells were seeded into Nunc 24-well plates as described above. The following day, plasmids were diluted in ultrapure water to a stock concentration of 200 ng/µl, and used to transfect cells using Lipofectamine™ LTX transfection reagent according to the manufacturer's instructions, with 500 ng of plasmids total per well. Plasmids were diluted to the required final concentration in OptiMEM (Gibco, ThermoFisher, USA), and cells were cultured in 450 µl DCCM1 starvation medium without penicillin and streptomycin. For detailed reporter construct information and sources see Table S2. The HRE reporter assay was comprised of 450 ng HRE plasmid and 50 ng *Renilla* luciferase plasmid per well, the latter acting as a constitutively activated control [90]. For the HIF-CAD reporter assay [29] (measuring FIH activity), 225 ng GAL4-DBD-HIF1α-CAD plasmid was added along with 225 ng UAS-luciferase plasmid, and 50 ng *Renilla* luciferase per well. After 16 h, the transfection medium was removed, and cells were exposed to PM in 600 µl of DCCM1 starvation medium for 24 h. Cells were washed once with ice-cold PBS, and harvested with passive lysis buffer (Promega, Wisconsin, USA) with gentle rocking for 15 min. Luciferase activity was then measured using a Dual-Luciferase^®^ Reporter Assay System kit (Promega) according to the manufacturer's instructions. All samples were tested in duplicate.

#### Statistical analysis

Unless otherwise stated in the respective figure legends, graphs were generated using GraphPad Prism v9.5.1(GraphPad Software, Inc. California, USA.) or R studio. The Shapiro–Wilk test was used to test for normality in data sets, with *p* ≤ 0.05 indicating skewed data. For normally distributed data, a one-way ANOVA was conducted with a Dunnett’s post-hoc test for pairwise analyses. For skewed data, Friedman’s test was used, followed by Dunn’s post-hoc test. For normally distributed data, bars represent the mean + standard error of the mean (SEM). For skewed data (and GSVA score graphs), boxplots show the median (central line), 25th and 75th percentiles (lower and upper box limits, respectively), and whiskers represent the range. P values ≤ 0.05 were considered statistically significant. In all figures, statistically significant values are indicated: * = *p* ≤ 0.05, ** = *p* ≤ 0.01, *** = *p* ≤ 0.001, **** = *p* ≤ 0.0001, ns = not significant. In all cases, N numbers reported in the figure legends represent individual donors where primary cells are used, or independent experiments using cultures of different passages where cell line cells are used.

## Supplementary Information

Additional file1

## Data Availability

The raw RNA-Seq data is available via the Gene Expression Omnibus, accessible at GSE286058. All other data are available in the main text or the supplementary materials.
