# Supplementary material for: Copper-enriched automotive brake wear particles perturb human alveolar cellular homeostasis
Source: Part Fibre Toxicol. 2025 Feb 13;22:4. doi: 10.1186/s12989-024-00617-2 (PMC11823208; doi:10.1186/s12989-024-00617-2)
Supplement: Supplementary file 1 — Additional file1 [file 12989_2024_617_MOESM1_ESM.pdf]

Supplementary Materials for

Copper-enriched automotive brake wear particles perturb human alveolar cellular homeostasis

James G. H. Parkin[*] *et al.*

*Corresponding author. Email: j.parkin@soton.ac.uk

This PDF file includes:

Figs. S1 to S4
Tables S1 to S2

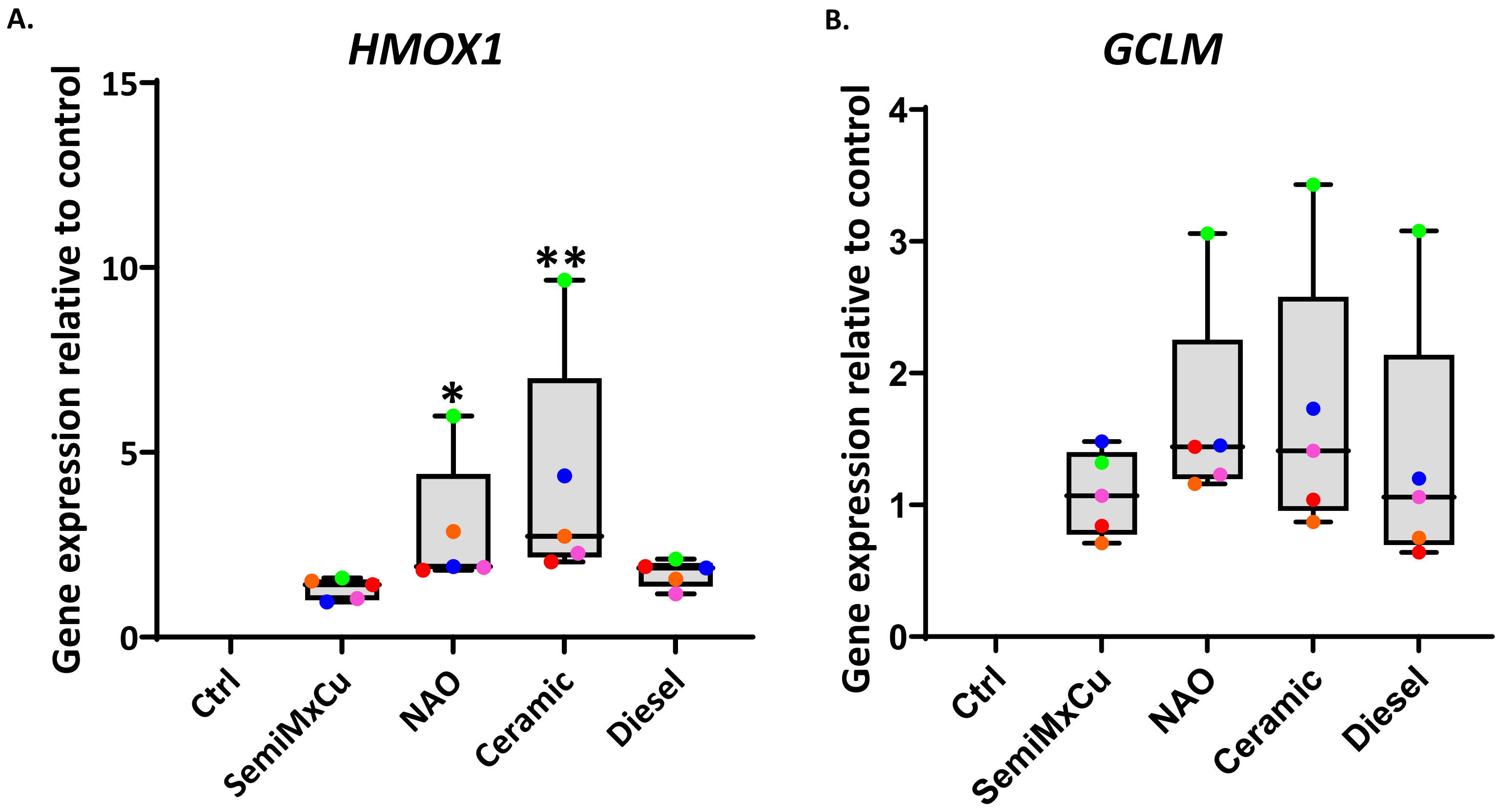

Fig. S1. Primary ATII cells were exposed to 8 µg/cm^2 of the 5 different PM types for 24 hours, after which gene expression was assessed using RT-qPCR. **A.** *HMOX1* gene expression. Determined via RT-qPCR. N = 5. **B.** *GCLM* gene expression. Determined via RT-qPCR. N = 5. Negative control represents cells that were not exposed to PM. As data was not normally distributed, a Friedman's test, along with a Dunn's post-hoc test was used to assess significant changes induced by PM compared to the control for both *HMOX1* and *GCLM*. Statistically significant values are indicated with the star notation on the graphs. * = P ≤ 0.05, ** = P ≤ 0.01.

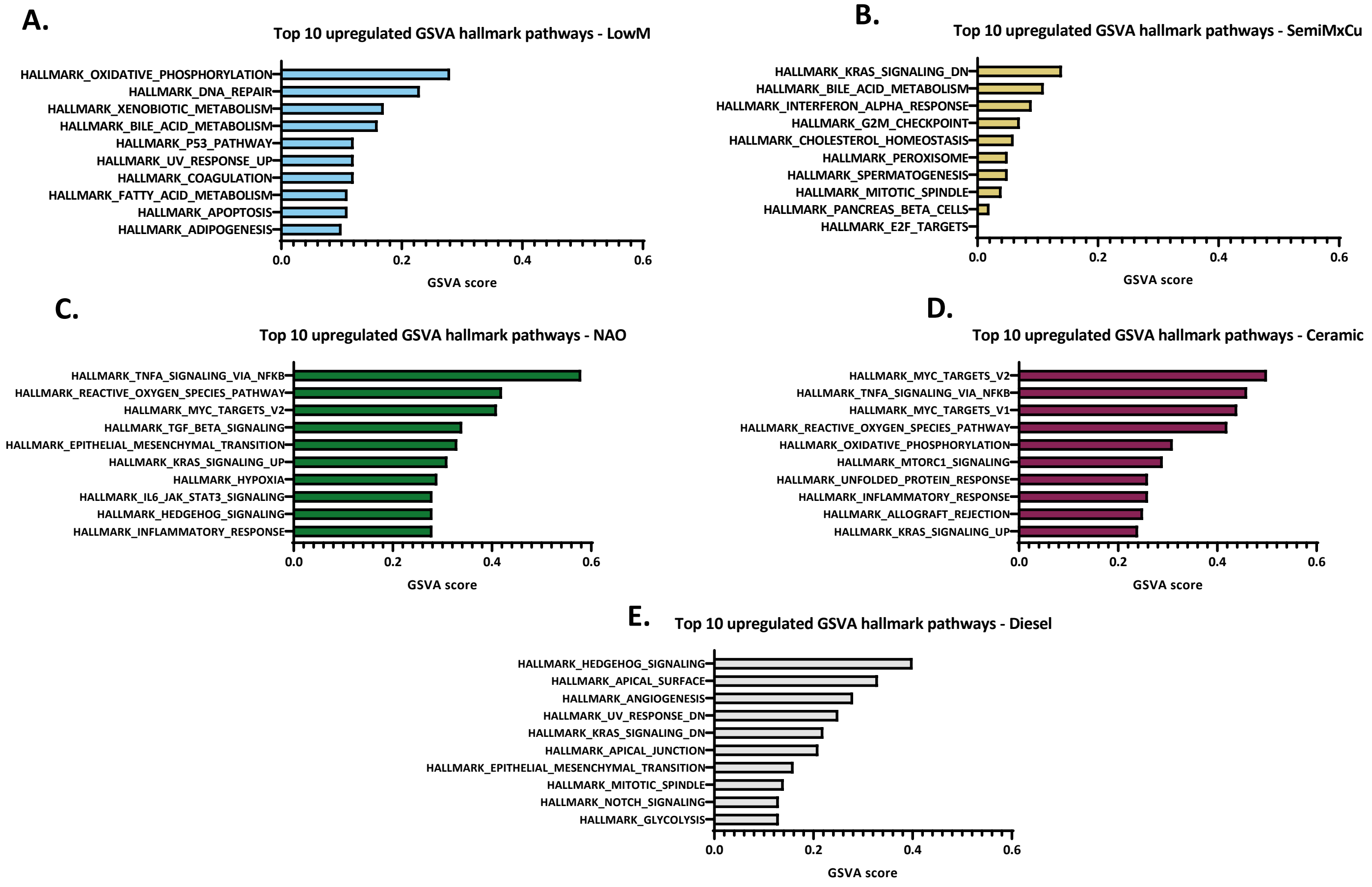

Fig. S2. ATII cells were exposed to 8 μg/cm^2 of the 5 different vehicle-derived PM types for 6 hours, after which bulk RNA-Seq was conducted. **A-E:** Gene Set Variation Analysis (GSVA) scores for the top 10 most upregulated hallmark pathways by induced by the respective PM types.

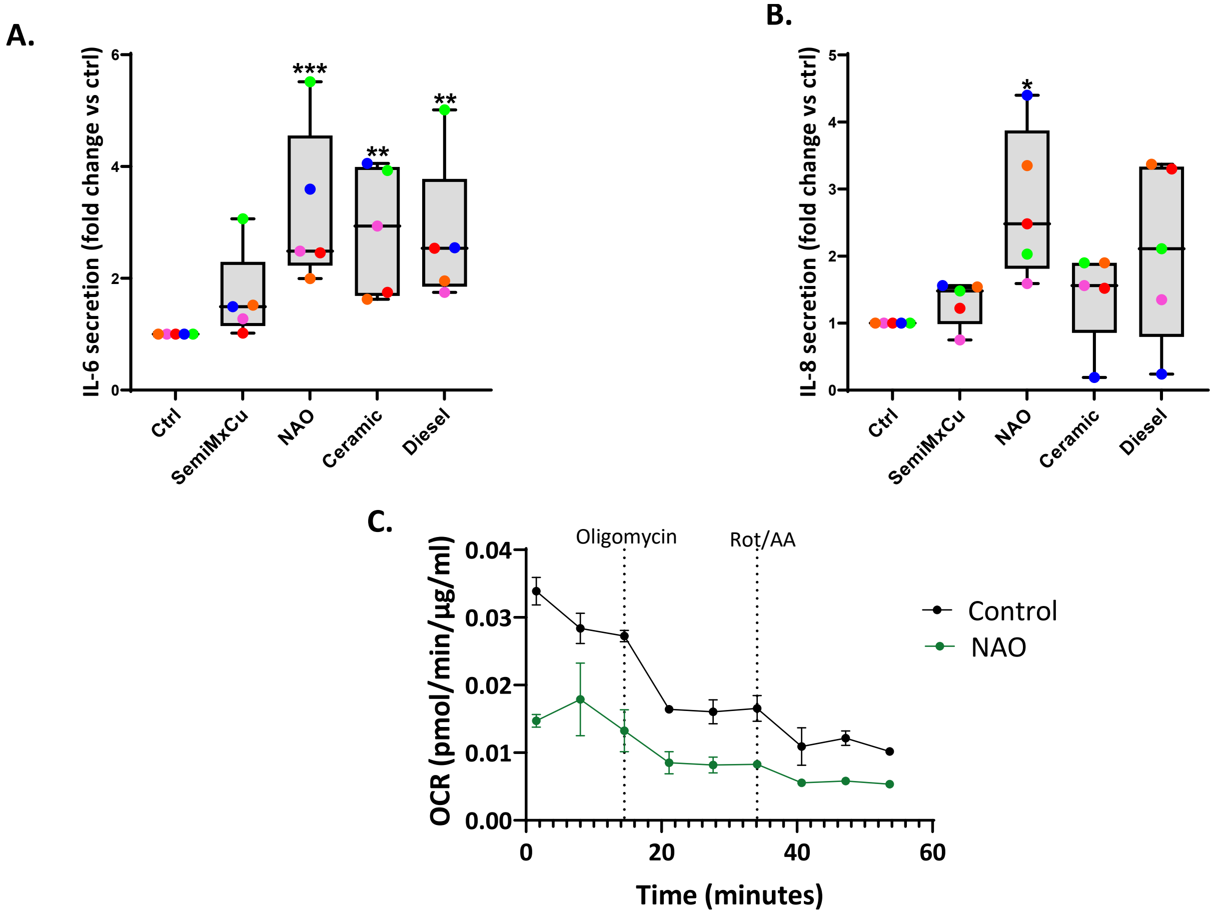

Fig. S3. ATII cells, and primary ATII cells were exposed to 8-32 µg/cm^2 of the 5 different PM types for 24 hours, after which various markers of inflammation were examined. **A.** Primary ATII cell IL-6 protein secretion was determined after exposure to 8 µg/cm^2 of the different PM types for 24 hours. Determined via ELISA. N = 5. **B.** Primary ATII cell IL-8 protein secretion was determined after exposure to 8 µg/cm^2 of the different PM types for 24 hours. Determined via ELISA. N = 5. **C.** Representative oxygen consumption rate over time from the ATP rate assay. Negative control represents cells that were not exposed to PM. In A and B, a Friedman's test was used followed by a Dunn's post-hoc test. Statistically significant values are indicated with the star notation on the graphs. * = P ≤ 0.05, ** = P ≤ 0.01, *** = P ≤ 0.001, **** = P ≤ 0.0001.

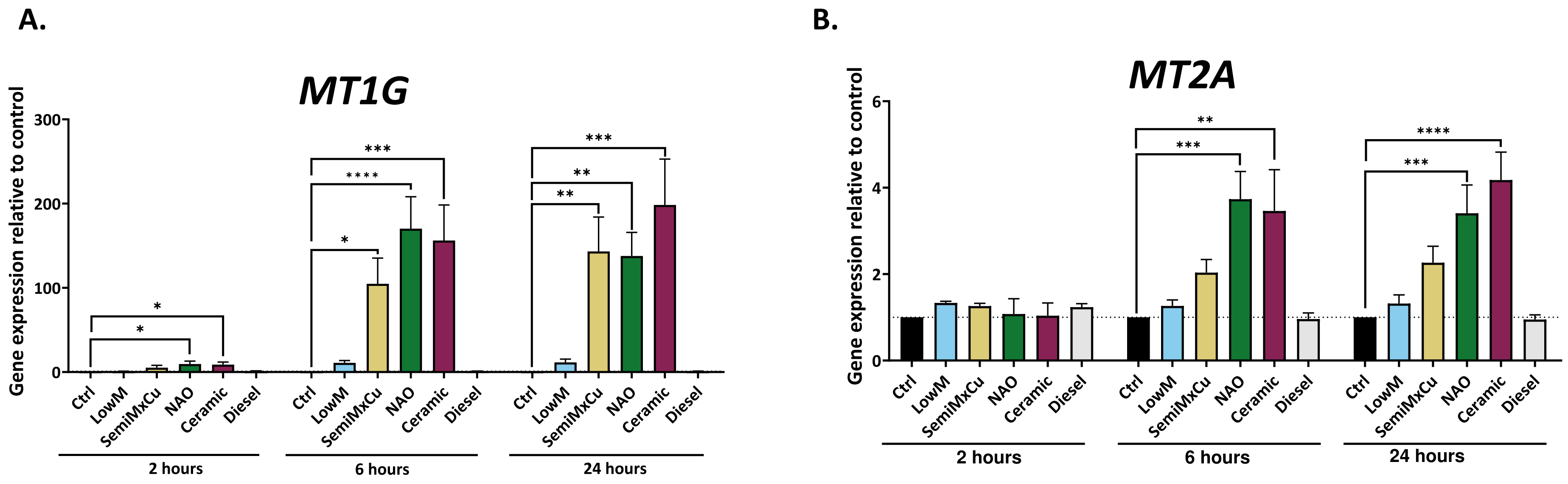

Fig. S4. ATII cells were exposed to 8 μg/cm^2 of the 6 different PM types for 2-24 hours, after which gene expression was assessed using RT-qPCR. **A.** ATII cell *Metallothionein 1G* (*MT1G*) expression was determined using RT-qPCR after exposure to 8 μg/cm^2 of the 5 different PM types, for 2, 6 and 24 hours, n = 5. **B.** ATII cell *Metallothionein 2A* (*MT2A*) expression was determined using RT-qPCR after exposure to 8 μg/cm^2 of the 5 different PM types, for 2, 6, and 24 hours. Data was represented as mean +SEM, and a RM 1W-ANOVA test was used with a Dunnett's post-hoc test. In E and F, Friedman's test was used followed by a Dunn's post-hoc test. Statistically significant values are indicated with the star notation on the graphs. * = P ≤ 0.05, ** = P ≤ 0.01, *** = P ≤ 0.001 **** = P ≤ 0.0001

Table S1. Elemental concentrations (ppm) of the vehicle-derived PM types in this study, determined by ICP-MS. %RSD for measurement given in brackets. LLoD = lower limit of detection, this was calculated as the mean elemental concentration of 5 distilled water samples + three times the standard deviation. bd = value below the LLoD.

	Element concentration, ppm (% RSD)					
Element	LowM	SemiMxCu	NAO	Ceramic	Diesel	LLoD
Li	0.6 (10.8)	1.369 (1.7)	0.759 (2.1)	1.378 (1.5)	0.318 (3.2)	0.29
B	93.5 (8.5)	14.68 (1.4)	13.988 (0.8)	20.17 (1.4)	bd	6.53
Na	5085 (3.6)	1590 (5.7)	1670 (5.9)	2300 (12.9)	1851.8 (0.9)	272.42
Mg	4940.5 (0.7)	1156.6 (0.7)	6287.6 (0.8)	10200 (1.5)	248.4 (1.3)	177.66
Al	4573.1 (0.8)	1042.9 (0.9)	3561.9 (0.8)	6256 (1.2)	bd	289.48
P	330.8 (1.6)	184.1 (2.0)	166.5 (2.7)	178.4 (2.5)	946.4 (2.1)	65.67
K	750.6 (1.2)	bd	12920 (1.1)	16606 (1.0)	609.1 (1.4)	293.13
Ca	7835 (2.4)	12450 (1.9)	15840 (1.9)	20630 (2.4)	2880 (5.2)	1968.39
Sc	0.3 (39.1)	9.84 (5.7)	219.5 (1.5)	356.3 (1.1)	0.3 (11.1)	0.05
Ti	bd	231.2 (2.1)	16340 (2.8)	21710 (2.9)	bd	142.86
V	19.64 (1.1)	37.68 (1.4)	9.559 (2.2)	17.25 (1.3)	bd	1.69
Cr	881.8 (2.8)	1654.9 (0.8)	211.6 (1.1)	357.8 (1.2)	13.633 (0.8)	2.22
Mn	717.37 (0.5)	2632.2 (0.9)	605.59 (1.0)	1126 (1.4)	9.299 (1.8)	1.62
Fe	98173 (0.7)	353890 (0.7)	92330 (1.0)	123490 (1.0)	611.67 (0.4)	106.95
Ni	65.778 (0.3)	109.01 (0.8)	101.98 (0.8)	170.1 (1.0)	12.07 (0.6)	8.3
Co	9.272 (1.3)	19.914 (0.6)	10.029 (0.8)	17.57 (1.5)	0.41 (6.0)	0.06
Cu	4406.2 (0.4)	926.2 (1.2)	55500 (1.0)	80886 (0.9)	221.34 (0.4)	7.73
Zn	2674 (0.7)	18270 (0.6)	1300 (15.7)	655.6 (1.1)	936.86 (0.4)	282.61
As	6.7666 (0.6)	25.984 (0.8)	6.802 (1.2)	9.4902 (0.7)	bd	1.55
Se	0.2 (28.0)	0.2 (16.1)	0.1 (22.3)	0.1 (28.1)	bd	0.03
Rb	3.549 (2.3)	bd	21.16 (1.6)	28.83 (2.3)	bd	0.57
Sr	48.105 (0.9)	497.41 (0.4)	446.49 (0.9)	538.1 (1.2)	bd	3.8
Y	0.22 (6.1)	6.458 (1.3)	79.8 (1.2)	111.9 (1.1)	0.13 (6.7)	0.05
Zr	45.9 (5.3)	1137.6 (0.8)	42170 (1.1)	57730 (1.4)	57.369 (0.8)	5.47
Nb	3.734 (1.1)	3.45 (1.4)	45.26 (1.1)	69.205 (1.0)	0.13 (4.3)	0.12
Mo	1593.3 (0.8)	46.53 (4.2)	78.116 (1.0)	58.58 (1.1)	8.595 (2.2)	0.2
Ag	0.312 (4.6)	0.895 (2.8)	17.08 (2.1)	28.42 (1.7)	0.1 (10.1)	0.04
Cd	0.23 (9.2)	0.248 (4.1)	0.4 (11.9)	0.5 (16.5)	0.57 (6.1)	0.02
Sn	2537.8 (0.6)	bd	6066 (1.5)	8160 (1.5)	bd	222.26
Cs	0.1 (18.9)	bd	0.3 (3.7)	0.427 (4.7)	bd	0.02
Ba	1257.5 (0.9)	20098 (0.4)	23041 (0.5)	32123 (0.5)	bd	12.12
La	0.181 (3.2)	1.0009 (0.7)	1.3827 (0.5)	1.4413 (0.5)	bd	0.11
Ce	0.735 (1.1)	1.71 (2.4)	3.154 (1.8)	4.085 (1.4)	1.742 (2.5)	0.27
Pr	0.05 (5.3)	0.175 (4.9)	0.313 (3.5)	0.3639 (0.7)	bd	0.03
Nd	0.18 (7.9)	0.665 (4.8)	1.226 (2.6)	1.56 (1.9)	bd	0.12
Gd	bd	0.35 (6.6)	1.513 (2.6)	1.708 (1.9)	bd	0.02
Yb	bd	1.66 (9.0)	17.52 (2.0)	19.35 (2.5)	bd	0.02
Hf	1.08 (6.1)	52.875 (0.5)	1074 (1.2)	1417 (1.5)	1.447 (1.7)	0.17
Tl	0.1 (20.5)	0.13 (9.6)	0.081 (4.5)	0.1 (19.3)	0.197 (3.1)	0.01
Pb	2.6395 (1.0)	7.7898 (0.8)	8.134 (1.4)	12.71 (1.5)	3.5073 (0.8)	2.22

Table S2. Key Resources Table

Reagent type (species) or resource	Designation	Source or reference	Identifiers	Additional information
transfected construct (*Homo-sapiens*)	GAL4DBD-HIF1αCAD (residues 652-826) used in HIF1αCAD reporter system	Ratcliffe lab (University of Oxford), Coleman *et al*(22)		
transfected construct (*Homo-sapiens*)	UAS-luc reporter used in HIF1αCAD reporter system	Ratcliffe lab (University of Oxford), Coleman *et a*(22)		
transfected construct (*Homo-sapiens*)	HRE luciferase reporter construct	Wang lab (University of Southampton), Zhao *et al*(*79*)		
transfected construct (*Homo-sapiens*)	*Renilla* luciferase control for reporter assays	Promega	E2261	pRL-CMV
cell line (*Homo-sapiens*)	ATII cells; type-II alveolar epithelial cells	Kemp *et al*(70), Coelho *et a*(66), Molina-Arcas *et al*(67), and Yao *et al*(68)		
antibody	Rabbit Anti-Mouse IG/HRP secondary antibody	Agilent	P0260	Western blot 1:1000
antibody	Purified Mouse Anti-Human HIF-1α	BD Biosciences	610958	Western blot 1:1000
antibody	Anti-β-Actin−Peroxidase antibody, Mouse monoclonal	Sigma	A3854	Western blot 1:50,000
chemical compound, drug	Dimethyloxalylglycine (DMOG)	Sigma	400091	
chemical compound, drug	N-[[1,2-Dihydro-4-hydroxy-2-oxo-1-(phenylmethyl)-3-quinolinyl]carbonyl]-glycine, N-[[4-Hydroxy-2-oxo-1-(phenylmethyl)-1,2-dihydro-3-quinolinyl]carbonyl]glycine (IOX2)	Selleckchem	S2919	
chemical compound, drug	Tetraethylene pentamine, technical grade	Sigma	T11509-100G	

Reagent type (species) or resource	Designation	Source or reference	Identifiers	Additional information
chemical compound, drug	N,N,N′,N′-Tetrakis(2-pyridylmethyl)ethylenediamine (TPEN)	Sigma	P4413-50MG	
commercial assay or kit	CytoTox 96® Non-Radioactive Cytotoxicity Assay	Promega	G1780	
commercial assay or kit	DuoSet® IL-6 ELISA kit	R&D systems	DY206	
commercial assay or kit	DuoSet® IL-8 ELISA kit	R&D systems	DY208	
commercial assay or kit	High-Capacity cDNA Reverse Transcription Kit	ThermoFisher	4368814	
commercial assay or kit	Lipofectamine™ LTX Reagent with PLUS™ Reagent	ThermoFisher	15338030	
commercial assay or kit	Micro BCA™ Protein Assay Kit	ThermoFisher	23235	
commercial assay or kit	Dual-Luciferase® Reporter Assay System	Promega	E1960	
commercial assay or kit	Seahorse XFp Real-Time ATP Rate Assay Kit	Agilent	103591-100	
commercial assay or kit	Seahorse XF HS Mini FluxPak	Agilent	103723-100	
commercial assay or kit	TaqMan™ Fast Advanced Master Mix	ThermoFisher	4444556	
commercial assay or kit	Monarch Total RNA Miniprep Kit	New England Biolabs	T2010S	
other	2′, 7′-Dichlorodihydrofluoroscin diacetate (H_2-DCF-DA)	Sigma	D6883	
other	3-(4,5-dimethylthiazol-2-yl)-2,5-diphenyltetrazolium bromide (MTT)	Sigma	M5655	
other	30% acrylamide/0.8% bis acrylamide	Geneflow	EC-890	
other	Ammonium persulphate (APS)	Pharmacia Biotech	17-1311-01	
other	L-Ascorbic acid	Sigma	A4544-25G	
other	Bromophenol blue	Sigma	B-5525	
other	Chelex®-100 resin	Sigma	C7901-25G	
other	Clarity western ECL substrate	Bio-Rad	170-5061	

Reagent type (species) or resource	Designation	Source or reference	Identifiers	Additional information
other	Complete protease inhibitor	Roche	04 693 116 001	
other	ROMIL-SpA™ concentrated hydrochloric acid	Romil	H396M	
other	ROMIL-SpA™ concentrated hydrofluoric acid	Romil	H405P	
other	ROMIL-SpA™ concentrated nitric acid	Romil	H566P	
other	DCCM1 without L-Glutamine Serum-Free Media	GeneFlow	K1-0502	
other	Deferoxamine mesylate salt	Sigma	D9533-1G	
other	Dimethyl sulfoxide (DMSO) (Sigma, USA, D2650)	Sigma	D2650	
other	Deoxyribonuclease 1 (DNase)	Sigma	DN25	
other	Foetal Bovine Serum, Heat Inactivated	ThermoFisher	A3840001	
other	Glycine	Sigma	G8898-1KG	
other	HBSS (+ calcium, + magnesium, no phenol red)	ThermoFisher	15266355	
other	HBSS (-calcium, -magnesium)	ThermoFisher	14170120	
other	L-Glutamine	ThermoFisher	25030081	
other	Marvel Milk Powder	N/A	N/A	
other	Methanol	Sigma	34860-2.5L-R	
other	N,N,N′,N′-Tetramethyl ethylenediamine (TEMED)	Sigma	T7024-25ML	
other	Newborn calf serum (heat inactivated; NCS)	Invitrogen	26010-074	
other	Nunc™ 24-well plates	ThermoFisher	142475	
other	Nunc™ 96-well plates	ThermoFisher	167008	
other	Penicillin/Streptomycin	ThermoFisher	15140122	
other	PhosSTOP	Roche	04 906 837 001	
other	Precision Plus Protein Kaleidoscope Std (10-250kDa)	BioRad	11610395	
other	Penicillin-streptomycin-glutamine (PSG)	Invitrogen	10378-016	
other	PVDF membranes	Bio-Rad	1620177	
other	Seahorse XF DMEM assay medium pack, pH 7.4	Agilent	103680-100	

Reagent type (species) or resource	Designation	Source or reference	Identifiers	Additional information
other	Sodium dodecyl sulphate	Sigma	L6026-1KG	
other	T25 cell culture flask	ThermoFisher	156340	
other	T75 cell culture flask	ThermoFisher	156472	
other	tert-Butyl hydroperoxide (TBHP) - Luperox® TBH70X	Sigma	458139	
other	Triton-X-100	Lab Planet	17-1315-01	
other	Trizma® base	Sigma	T1503-25G	
other	Trypan blue	Sigma	T8154-100ML	
other	Trypsin-EDTA (0.25%)	ThermoFisher	25200056	
other	B-mercaptoethanol	Sigma	M7522-100ML	
sequence-based reagent	TaqMan™ Gene Expression Assay (FAM)	ThermoFisher	4331182	
sequence-based reagent	TaqMan™ Gene Expression Assay (VIC)	ThermoFisher	4448489	
